# A Mutation in the *SUV39H2* Gene in Labrador Retrievers with Hereditary Nasal Parakeratosis (HNPK) Provides Insights into the Epigenetics of Keratinocyte Differentiation

**DOI:** 10.1371/journal.pgen.1003848

**Published:** 2013-10-03

**Authors:** Vidhya Jagannathan, Jeanette Bannoehr, Philippe Plattet, Regula Hauswirth, Cord Drögemüller, Michaela Drögemüller, Dominique J. Wiener, Marcus Doherr, Marta Owczarek-Lipska, Arnaud Galichet, Monika M. Welle, Katarina Tengvall, Kerstin Bergvall, Hannes Lohi, Silvia Rüfenacht, Monika Linek, Manon Paradis, Eliane J. Müller, Petra Roosje, Tosso Leeb

**Affiliations:** 1 Institute of Genetics, Vetsuisse Faculty, University of Bern, Bern, Switzerland; 2 DermFocus, University of Bern, Bern, Switzerland; 3 Division of Clinical Dermatology, Vetsuisse Faculty, University of Bern, Bern, Switzerland; 4 Division of Neuroscience, Vetsuisse Faculty, University of Bern, Bern, Switzerland; 5 Institute of Animal Pathology, Vetsuisse Faculty, University of Bern, Bern, Switzerland; 6 Veterinary Public Health Institute, Vetsuisse Faculty, University of Bern, Bern, Switzerland; 7 Science for Life Laboratory, Department of Medical Biochemistry and Microbiology, Uppsala University, Uppsala, Sweden; 8 Department of Clinical Sciences, Swedish University of Agricultural Sciences, Uppsala, Sweden; 9 Research Programs Unit, Molecular Neurology, University of Helsinki, Helsinki, Finland; 10 Department of Veterinary Biosciences and Department of Medical Genetics, University of Helsinki, Helsinki, Finland; 11 Folkhälsan Institute of Genetics, Helsinki, Finland; 12 Dermavet, Tierklinik Aarau-West, Oberentfelden, Switzerland; 13 Tierärztliche Spezialisten, Hamburg, Germany; 14 Department of Clinical Sciences, Faculté de Médecine Vétérinaire, University of Montreal, St-Hyacinthe, Québec, Canada; Uppsala University, Sweden

## Abstract

Hereditary nasal parakeratosis (HNPK), an inherited monogenic autosomal recessive skin disorder, leads to crusts and fissures on the nasal planum of Labrador Retrievers. We performed a genome-wide association study (GWAS) using 13 HNPK cases and 23 controls. We obtained a single strong association signal on chromosome 2 (p_raw_ = 4.4×10^−14^). The analysis of shared haplotypes among the 13 cases defined a critical interval of 1.6 Mb with 25 predicted genes. We re-sequenced the genome of one case at 38× coverage and detected 3 non-synonymous variants in the critical interval with respect to the reference genome assembly. We genotyped these variants in larger cohorts of dogs and only one was perfectly associated with the HNPK phenotype in a cohort of more than 500 dogs. This candidate causative variant is a missense variant in the *SUV39H2* gene encoding a histone 3 lysine 9 (H3K9) methyltransferase, which mediates chromatin silencing. The variant c.972T>G is predicted to change an evolutionary conserved asparagine into a lysine in the catalytically active domain of the enzyme (p.N324K). We further studied the histopathological alterations in the epidermis *in vivo*. Our data suggest that the HNPK phenotype is not caused by hyperproliferation, but rather delayed terminal differentiation of keratinocytes. Thus, our data provide evidence that SUV39H2 is involved in the epigenetic regulation of keratinocyte differentiation ensuring proper stratification and tight sealing of the mammalian epidermis.

## Author Summary

Hereditary nasal parakeratosis (HNPK), an inherited monogenic autosomal recessive skin disorder, leads to crusts and fissures on the nasal planum of Labrador Retrievers. We performed a genome-wide association study (GWAS) and mapped the causative mutation to a small interval on chromosome 2. We re-sequenced the entire genome of one HNPK affected dog and detected three protein-changing mutations in the critical interval. We genotyped these three variants in larger cohorts of dogs and only one showed perfect genotype-phenotype association, prompting us to consider this variant, a missense mutation in SUV39H2, as the most likely causative variant for HNPK. SUV39H2 is an enzyme involved in histone methylation and epigenetic silencing, a process determining the regulation of gene expression during keratinocyte differentiation. A loss of SUV39H2 function is predicted to result in delayed differentiation, which is compatible with the histopathological changes that we observed in biopsies from nasal epidermis of HNPK affected Labrador Retrievers. Thus, our results provide the most likely causative mutation for a hereditary disease in dogs that can now be eradicated by selective breeding. In addition, our results also provide the first hint that SUV39H2, whose *in vivo* function is not fully understood, may be involved in keratinocyte differentiation.

## Introduction

The outermost layer of the skin, the epidermis, is a stratified squamous epithelium which forms a barrier against the environment. It is renewed throughout lifetime by continuous proliferation of keratinocytes in the lower layers of the epithelium juxtaposed to a basement membrane. Once the cells exit the cell cycle, they start to move outwards and go on to differentiate while building up the stratified architecture of the epidermis. Epidermal renewal is a tightly controlled process that relies on well-orchestrated and finely tuned changes in the expression of genes encoding cell cycle regulators and structural components such as keratins or adhesion molecules. In their final stage of terminal differentiation, the keratinocytes lose their nuclei and cytoplasmic organelles to become corneocytes, which form the outermost tightly sealing layer of the epidermis. Corneocytes from the surface of the epidermis will eventually be sloughed into the environment. A complete turnover of the nonglabrous epidermis takes about 40–56 days in healthy humans, 8–10 days in mice and about 22 days in dogs [1], [2].

Much progress has been made in the understanding of gene expression in keratinocytes during all stages of differentiation and a number of involved signaling pathways are now known [3]–[5]. The overall differentiation program of keratinocytes follows a cline of very active global transcription in the basal cells towards more and more restricted transcription profiles in further differentiated cells. There is increasing evidence that epigenetic processes such as DNA methylation and histone modifications play an important role in the gradual transcriptional silencing during keratinocyte differentiation [6].

Spontaneous animal mutants with hereditary defects in the normal differentiation of the epidermis provide an opportunity to identify further components of the complex regulatory network required to establish specific patterns of gene expression. Due to their special population structure purebred dogs are particularly well suited for genetic analysis [7]. Successful examples for the utilization of dog genetics in skin research include the identification of genes involved in the determination of hair characteristics [8], ectodermal development [9], one form of ichthyosis [10], congenital keratoconjunctivitis sicca and ichthyosiform dermatosis [11], and the excessive skin folding in Chinese Shar Pei dogs [12].

Hereditary nasal parakeratosis (HNPK) of Labrador Retrievers has been reported to consist of a defect in the differentiation of the specialized epidermis of the nasal planum [13], [14]. HNPK affected dogs develop crusts and fissuring of the nasal planum at a young age but are otherwise healthy. The pathognomonic histopathological changes consist of a marked diffuse parakeratotic hyperkeratosis characterized by the retention of nuclei in the stratum corneum and an accumulation of proteinaceous fluid (“serum lakes”) within the stratum corneum (Fig. S1). HNPK is inherited as a monogenic autosomal recessive trait and has so far exclusively been described in the Labrador Retriever breed [13].

In this study we used a genome-wide association study (GWAS) and whole genome re-sequencing approach (WGS) of an affected Labrador Retriever to identify the genetic lesion responsible for HNPK.

## Results

### Mapping of the causative mutation

**Figure 1:**
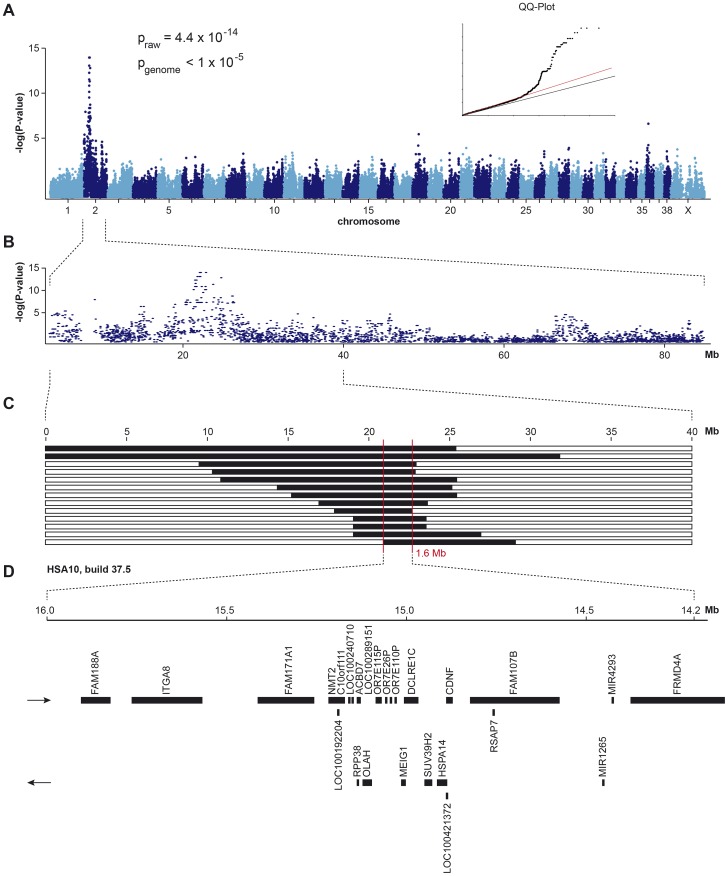
Mapping of HNPK in Labrador Retrievers. (A) A genome-wide association study using 13 cases and 23 controls indicates a strong signal with multiple associated SNPs on CFA 2. (B) The detailed view of CFA 2 delineates an associated interval of ∼4 Mb. (C) Homozygosity mapping. Each horizontal bar corresponds to one of the 13 analyzed cases. Homozygous regions with shared alleles are shown in black. A shared homozygous interval of ∼1.6 Mb delineates the exact boundaries of the critical interval from 20,818,258–22,414,948 bp (CanFam 3.1 assembly). (D) Gene content of the corresponding human interval on HSA 10 (NCBI annotation, genome build 37.5).

We collected samples from 13 HNPK affected Labrador Retrievers and 23 controls and genotyped them with the 173 k SNP chip. After removing 66,981 markers, which had low call rates (<90%), were non-informative (MAF<0.05), or showed a strong deviation from Hardy-Weinberg equilibrium in the controls (p<10^−5^), we retained 106,681 markers for the final genome-wide allelic association study. Three best-associated SNPs in the GWAS had identical raw p-values of 4.4×10^−14^ (Figure 1A). The corrected p-value after 100,000 permutations was <10^−5^. The 92 best-associated SNPs with raw p-values of less than 5×10^−7^ were all located on chromosome 2 (CFA 2, Figure 1B). The genomic inflation factor in this analysis was 1.28.

Subsequently, we applied a homozygosity mapping approach to fine-map the region containing the HNPK mutation. We hypothesized that the affected dogs most likely were inbred to one single founder animal. In this scenario, the affected individuals were expected to be identical by descent (IBD) for the causative mutation and flanking chromosomal segments. We analyzed the cases for extended regions of homozygosity with simultaneous allele sharing. Two genome regions, of which the larger one coincided with the associated interval on CFA 2, fulfilled our search criteria. In this interval on CFA 2, all 13 affected dogs were homozygous and shared identical alleles over 110 consecutive SNP markers. We concluded that the causative mutation should be located in the ∼1.6 Mb critical interval between the closest heterozygous markers on either side of the homozygous segment (CFA2:20,818,258–22,414,948, CanFam 3.1 assembly; Figure 1C).

### Mutation identification

A total of 15 genes and loci are annotated in the critical interval on CFA 2 (NCBI CanFam 3.1 annotation). In the corresponding human interval with its presumably more complete annotation 25 genes and loci are contained (Figure 1D). In order to obtain a comprehensive overview of all variants in the critical interval we sequenced the whole genome of one affected Labrador Retriever. We collected 502 million 2×100 bp paired-end reads from a shotgun fragment library corresponding to roughly 38× coverage of the genome. We called SNPs and indel variants with respect to the reference genome of a presumably non-affected Boxer. Across the entire genome, we detected ∼3 million homozygous variants (Table 1). Within the critical interval there were 1,533 variants, of which 4 were predicted to be non-synonymous (Table 2). One of these variants turned out to represent an artifact due to an error in the reference genome assembly.

**Table 1:** Variants detected by whole genome re-sequencing of an affected Labrador Retriever.

Filtering step	Number of variants
Variants in the whole genome^a^	2,980,294
Variants in the critical 1.6 Mb interval on CFA 2	1,533
Non-synonymous variants in the whole genome^a^	7,911
Non-synonymous variants in the critical 1.6 Mb interval on CFA 2	4

a The sequences were compared to the reference genome (CanFam 3.1) from a Boxer. Only variants that were homozygous in the affected Labrador Retriever are reported.

**Table 2:** Four non-synonymous variants in the critical interval of an HNPK affected Labrador Retriever with respect to the Boxer reference genome (CanFam 3.1).

Position on CFA 2	Reference allele	Variant allele	Gene	Variant (cDNA)	Variant (protein)
21,098,589	A	G	*ITGA8*	c.362A>G	p.K121R
21,584,989	C	-	*NMT2*	c.254delC^a)^	frameshift^a)^
21,612,666	T	C	*RPP38*	c.380A>G	p.E127G
21,731,842	A	C	*SUV39H2*	c.972T>G	p.N324K

a Subsequent analyses of the original Sanger reads from the dog genome project revealed that this is not a true variant, but rather an error in the reference genome assembly (data not shown).

**Figure 2:**
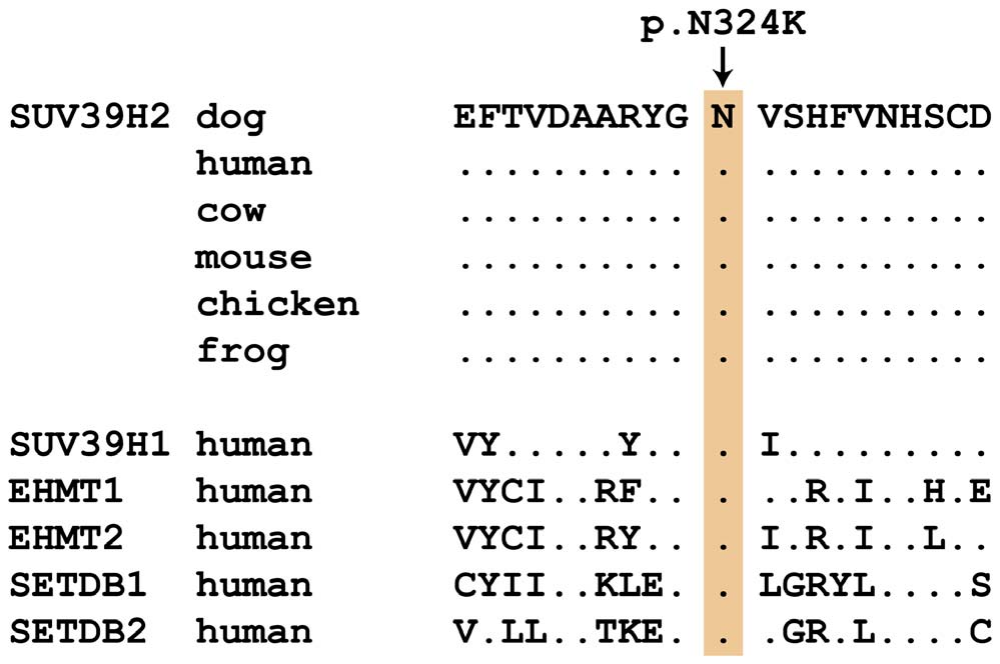
Evolutionary conservation of the asparagine residue at position 324 in the SUV39H2 protein. Position 324 within the catalytically active SET domain is perfectly conserved across vertebrates in all known SUV39H2 orthologs. In the lower part of the alignment the sequences of closely related paralogous histone methyltransferases with similar substrate specificity also demonstrate conservation of the asparagine at this position. The sequences were derived from the following database accessions: *C. lupus* SUV39H2 XP_535179.2, *H. sapiens* SUV39H2 NP_001180353.1, *B. taurus* SUV39H2 NP_001032556.1, *M. musculus* Suv39h2 NP_073561.2, *G. gallus* SUV39H2 NP_001026541.1, *X. laevis* suv39h2 NP_001091337.1, *H. sapiens* SUV39H1 NP_003164.1, *H. sapiens* EHMT1 NP_079033.4, *H. sapiens* EHMT2 NP_006700.3, *H. sapiens* SETDB1 NP_001138887.1, *H. sapiens* SETDB2 NP_114121.2.

We genotyped the three remaining non-synonymous variants in larger cohorts of dogs (Table 3). Only one variant, *SUV39H2:c.972T>G* remained perfectly associated in a very large cohort of more than 500 dogs. As expected for the causal variant, we found it exclusively in Labrador Retrievers, but not in 139 dogs from diverse other breeds. The *SUV39H2:c.972T>G* variant represents a missense mutation in the *SUV39H2* gene, encoding the “suppressor of variegation 3-9 homolog 2 (Drosophila)”, a histone 3 lysine 9 (H3K9) methyltransferase. The variant results in the change of an asparagine residue in the catalytically active SET domain to a lysine (p.N324K). The SET domain has been named according to the first proteins, in which it has been identified, Suvar(3)9, enhancer of zeste, and trithorax. SIFT and Polyphen-2 predict that the p.N324K variant affects protein function [15], [16]. The asparagine at position 324 is highly conserved across all known SUV39H2 orthologs and even across many other related H3K9 methyltransferases (Figure 2).

**Table 3:** Association of non-synonymous variants with the HNPK phenotype.

Genotype	Labrador Retriever cases	Labrador Retriever non-affected carriers^a^	Labrador Retriever controls	Dogs from other breeds
*ITGA:c.363A>G*				
*A/A*	-	-	1	2
*A/G*	-	1	2	2
*G/G*	15	3	5	10
*RPP38:c.381A>G*				
*A/A*	-	-	216	40
*A/G*	-	4	65	21
*G/G*	16	-	1	16
***SUV39H2:c.972T>G***				
***T/T***	**-**	**-**	**327**	**139**
***T/G***	**-**	**4**	**48**	**-**
***G/G***	**26**	**-**	**-**	**-**

a Parents of affected dogs were classified as obligate carriers.

### *In vivo* analysis of keratinocyte proliferation and differentiation

**Figure 3:**
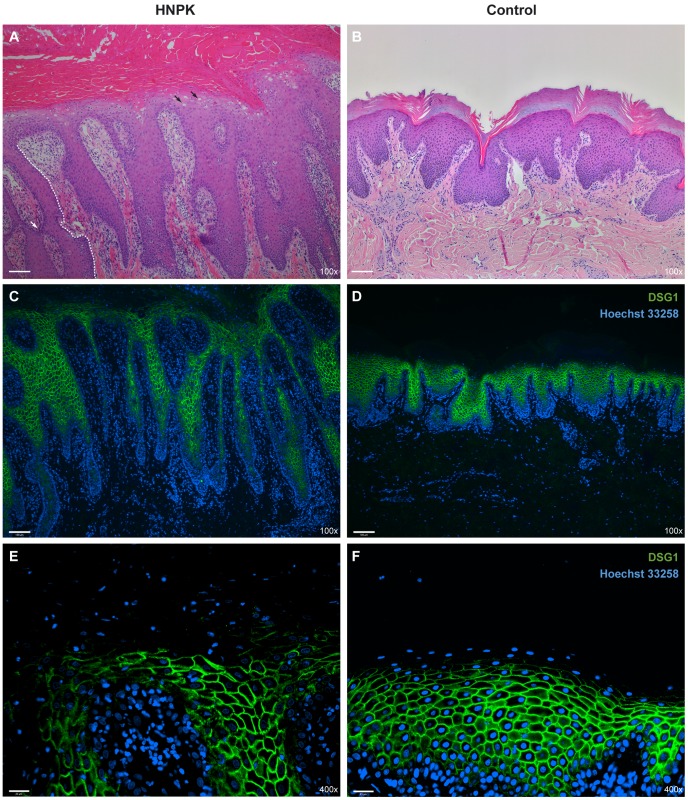
Nasal planum tissue of an HNPK affected and a non-affected Labrador Retriever. (A) Hematoxylin and eosin (HE) staining of an HNPK affected dog shows diffuse parakeratotic hyperkeratosis, hydropic keratinocytes in upper epidermal layers (black arrows), epidermal lymphocytic exocytosis, and a perivascular dermal lymphocytic infiltrate. The formation of elongated and sometimes fused slim rete pegs in the affected dog contributes to the thickening of the epidermis (the boundary of one rete peg is indicated by a white dashed line, one fusion is indicated by the white arrow). (B) HE staining of a control dog. (C–F) Immunodetection of desmoglein 1 (DSG1, green) in two different magnifications. Nuclei are counterstained with Hoechst 33258 (blue). No visible differences in the DSG1 staining patterns are present between HNPK affected and control dog. Scale bar = 100 µm for HE/DSG1 100× and 25 µm for DSG1 400×.

We re-investigated the histopathological changes in the nasal planum of HNPK affected Labrador Retrievers. The changes in hematoxylin and eosin (HE) stained sections were compatible with earlier descriptions [13], [14]. The nasal epidermis of affected dogs was covered with abundant parakeratotic keratin with an accumulation of numerous vacuoles or lakes filled with proteinaceous fluid. In addition to the lesions in the corneal layer, we noticed degenerative changes in the viable keratinocytes of the epidermis, such as striking cytoplasmic vacuolation (hydropic degeneration). Nonspecific changes, such as the presence of inflammatory cells, predominantly lymphocytes, were also present in the dermis and epidermis (Fig. 3A).

In addition to the re-examination of the previously published histopathological changes, we analyzed whether keratinocyte proliferation is altered in HNPK affected dogs. We performed quantitative immunofluorescence microscopy analyses for the proliferation marker Ki67 in paraffin sections of the nasal planum of 6 HNPK affected dogs and 6 non-affected control Labrador Retrievers. The number of Ki67 positive cells was not significantly increased in HNPK dogs compared with controls. There was no significant difference in the overall hyperproliferation and hyperplasia indices (p = 0.63 and 0.62, respectively). The ratio of Ki67 positive cells in the basal layer to Ki67 positive total cells was also comparable between groups (p = 0.55). This analysis confirmed that HNPK is not due to hyperproliferation of keratinocytes.

We also examined the expression pattern of the differentiation marker desmoglein 1 (DSG1). The expression of this adhesion molecule extends from the suprabasal layer to the last viable layers of the epidermis [17], [18]. Based on immunofluorescence analysis the DSG1 staining pattern was similar between HNPK affected and control Labrador Retrievers suggesting that a hypothetical change should primarily affect the differentiation process in the outermost layers of the epidermis (Fig. 3 C–F).

## Discussion

In this study we identified a missense variant of SUV39H2 as candidate causative genetic defect for HNPK in Labrador Retrievers. It has to be cautioned that our analysis focused on non-synonymous variants. We thus cannot formally rule out the possibility that a synonymous or non-coding regulatory variant in absolute linkage disequilibrium with the *SUV39H2:c.972T>G* variant is the actual causative variant. However, the strictly recessive mode of inheritance suggests a complete loss-of-function allele, which excludes a large fraction of regulatory variants with minor effects. It also has to be considered that our variant detection relied on short read mapping to an imperfect reference genome. Thus, we will have missed variants, which are located in genome segments that are not contained in the reference genome, such as gap regions. We may also have missed non-synonymous variants in genes that are not or not correctly annotated in the dog reference genome.

The identified missense variant is actually within a plausible functional candidate gene. SUV39H2 catalyzes the addition of methyl groups to the lysine 9 residue of histone 3 (H3K9) and transforms an unmethylated H3K9 into trimethylated H3K9me3 [19]. Methylation of H3K9 is a chromatin modification inducing transcriptional silencing and a typical hallmark of heterochromatin [20]. The crystal structure of SUV39H2 has been solved and the catalytically active site for the methyltransferase activity is located within the so-called SET domain [21]. As the SUV39H2:p.N324K variant in HNPK affected Labrador Retrievers affects a highly conserved amino acid residue within this SET domain it is conceivable that this variant abolishes SUV39H2 function completely or to a large extent.

While there is extensive knowledge on the biochemical activity of SUV39H2 and its role in heterochromatin formation, its specific physiological function *in vivo* is much less clear. Addressing this question has been difficult as mammals express a large number of closely related histone modifying enzymes including eight genes encoding specific H3K9 methyltransferases [21]. *Suv39h2* deficient mice and mice deficient in the closely related *Suv39h1* do not display any obvious phenotype [22]. However, alterations in the nasal planum have not been specifically evaluated in these mice.

In contrast, the double knockout mice deficient in both *Suv39h1* and *Suv39h2* exhibit severe deficiencies in genome stability and regulation of telomere length. Consequently, the double knockout mice have a greatly impaired viability and delayed growth [22], [23]. These data suggest that *Suv39h2* deficient mice do not display an overt phenotype as the *Suv39h2* function may be replaced by other redundant H3K9 methyltransferases.

The clinical symptoms of HNPK in Labrador Retrievers are exclusively restricted to the epidermis of the nasal planum. It is currently unclear why a defect in a broadly expressed histone modifying enzyme leads to a very tissue-specific clinical phenotype. We speculate that this may be due to the tissue-specific expression profiles of the partially redundant H3K9 methyltransferases, with the epidermis of the nasal planum being the only tissue where the loss of SUV39H2 cannot be compensated. We also cannot explain at this point why *Suv39h2* deficient mice do not display a similar phenotype. However, it can be argued that the nasal planum of mice is extraordinarily small and that subtle alterations are very difficult to assess.

Our morphological analyses clearly demonstrate that the HNPK phenotype is not due to hyperproliferation of keratinocytes. Thus, it seems highly probable that the HNPK phenotype is caused by an altered terminal keratinocyte differentiation. Similar expression patterns of the differentiation marker DSG1 in cases and control dogs provide further evidence that this delay in differentiation might be primarily localized in the outermost layers of the epidermis. SUV39H2, a chromatin modifying enzyme, is a logical candidate for being involved in the regulation of cell differentiation. Although we do not yet have complete functional proof for its role in keratinocyte differentiation, our genetic data together with the detailed *in vivo* histopathological analyses strongly suggest that SUV39H2:p.N324K is the causative defect for HNPK. Further studies with additional differentiation markers and RNA-seq experiments will help to clarify which downstream effector molecules are altered by the lacking chromatin modification in SUV39H2 deficient cells resulting in an impaired keratinocyte differentiation process.

In conclusion, we have identified a missense variant in the histone methylase SUV39H2 as most likely causative for HNPK in Labrador Retrievers. This provides a first indication of an involvement of this particular enzyme in the epigenetic regulation of keratinocyte differentiation.

## Materials and Methods

### Ethics statement

All animal experiments were performed according to the local regulations. The dogs in this study were examined with the consent of their owners. The study was approved by the “Cantonal Committee For Animal Experiments” (Canton of Bern; permits 22/07 and 23/10).

### Animal selection

We collected EDTA blood samples from Labrador Retrievers. We specifically used 26 cases and 164 controls, which could be unambiguously phenotyped based on photographs or direct inspection by veterinarians during the collection of the LUPA atopic dermatitis cohort [24]. We also used other DNA samples that were collected for various research projects at the Institute of Genetics of the University of Bern. For these other samples a non-affected phenotype was assumed (“population controls”) as HNPK is a young and relatively rare trait.

### DNA samples and SNP genotyping

We isolated genomic DNA from EDTA blood samples with the Nucleon Bacc2 kit (GE Healthcare). Genotyping was done on illumina canine_HD chips containing 173,662 SNP markers either at the Centre National de Génotypage, Evry, France or at the NCCR Genomics Platform of the University of Geneva. Genotypes were stored in a BC/Gene database version 3.5 (BC/Platforms).

### Genome-wide association study (GWAS) and homozygosity mapping

We used PLINK v1.07 [25] to perform genome-wide association analyses (GWAS). All 13 cases and 23 controls used for the analysis had call rates >90%. We removed 12,165 markers with call rates <90% from the analysis. We also removed 60,064 markers with minor allele frequency (MAF) <5% and 1 marker strongly deviating from Hardy-Weinberg equilibrium (p<10^−5^). The final dataset consisted of 36 dogs and 106,681 SNPs. We performed an allelic association study and determined an empirical significance threshold by performing 100,000 permutations of the dataset with arbitrarily assigned phenotypes. We also used PLINK to search for extended intervals of homozygosity with shared alleles as described previously [26]. Briefly, we looked for homozygous segments larger than 1 Mb and covering more than 100 consecutive markers. Two regions in the genome on CFA 2 (1.6 Mb shared homozygous interval) and CFA 11 (1.1 Mb shared homozygous interval) fulfilled the search criteria.

### Gene analysis

We used the dog CanFam 3 and the human 37 assemblies for all analyses. We used BLASTN searches to define the orthologous human chromosomal regions corresponding to the associated interval on CFA 2. For the candidate gene inspection we used the human annotation provided by NCBI (build 37.5). All numbering within the canine *SUV39H2* gene corresponds to the accessions XM_535179.3 (mRNA) and XP_535179.2 (protein). We analyzed the functional effects of variants *in silico* with SIFT and Polyphen-2 [15], [16].

### Whole genome sequencing of an affected Labrador Retriever

We prepared a fragment library with 300 bp insert size and collected three lanes of illumina HiSeq2000 paired-end reads (2×100 bp). We obtained a total of 502,733,347 paired-end reads or roughly 38× coverage. We mapped the reads to the dog reference genome using the Burrows-Wheeler Aligner (BWA) version 0.5.9-r16 [27] with default settings and obtained 438,768,718 uniquely mapping reads. After sorting the mapped reads by the coordinates of the sequence and merging the 3 lanes of data with Picard tools, we labeled the PCR duplicates also with Picard tools (http://sourceforge.net/projects/picard/). We used the Genome Analysis Tool Kit (GATK version v2.3–6, [28]) to perform local realignment and to produce a cleaned BAM file. Variant calls were then made with the unified genotyper module of GATK. Variant data for each sample were obtained in variant call format (version 4.0) as raw calls for all samples and sites flagged using the variant filtration module of GATK. Variant calls that failed to pass the following filters were labeled accordingly in the call set: (i) Hard to Validate MQ0≥4 & ((MQ0/(1.0 * DP)) >0.1); (ii) strand bias (low Quality scores) QUAL<30.0 ∥ (Quality by depth) QD<5.0 ∥ (homopolymer runs) HRun>5 ∥ (strand bias) SB>0.00; (iii) SNP cluster window size 10. The snpEFF software [29] together with the CanFam 3.1 annotation was used to predict the functional effects of detected variants. We considered the following snpEFF categories of variants as non-synonymous: NON_SYNONYMOUS_CODING, CODON_DELETION, CODON_INSERTION, CODON_CHANGE_PLUS_CODON_DELETION, CODON_CHANGE_PLUS_CODON_INSERTION, FRAME_SHIFT, EXON_DELETED, START_GAINED, START_LOST, STOP_GAINED, STOP_LOST, SPLICE_SITE_ACCEPTOR, SPLICE_SITE_DONOR. The critical interval contained 1,596,690 bp and 14,335 coding nucleotides, respectively. In our re-sequencing data, we had ≥4x coverage on 1,588,388 bp of the critical interval (99.5%) and all of the coding bases.

Additionally, we searched for structural variations (deletions, insertions, inversions) within the critical interval using the software Pindel [30]. This analysis identified 59 deletions and insertions between 7 and 687 bp in size in the critical interval. Most of these variants were within microsatellite repeats and none of them affected an exon of the annotated genes in the critical interval. A visual inspection of the paired-end read data in the integrative genomics viewer (IGV, [31]) also did not indicate any structural variation involving an annotated exon.

### Sanger sequencing

We used Sanger sequencing to confirm the illumina sequencing results and to perform targeted genotyping for selected variants. For these experiments we amplified PCR products using AmpliTaqGold360Mastermix (Applied Biosystems). PCR products were directly sequenced on an ABI 3730 capillary sequencer (Applied Biosystems) after treatment with exonuclease I and shrimp alkaline phosphatase. We analyzed the Sanger sequence data with Sequencher 5.1 (GeneCodes).

### Hematoxylin and eosin (HE) and immunofluorescence (IF) staining

Biopsies of the nasal planum of HNPK affected (*n* = 6) and non-affected control (*n* = 6) Labrador Retrievers were collected, fixed in 4% formalin and embedded in paraffin. Paraffin slides were generated and HE stained by standard methods. The immunofluorescence staining of Ki67 (official gene symbol *MKI67*) and DSG1 was carried out as described previously [32]. Briefly, the tissue was deparaffinized, followed by heat-mediated antigen retrieval in sodium citrate buffer, pH 6 (3×5 min at 720 W in a microwave). Slides were transferred into a moisture chamber, blocked with 4% bovine serum albumin and 5% normal goat serum in phosphate buffered saline containing MgCl_2_ and CaCl_2_ (“PBS^+^”) for 90 min at room temperature and incubated with primary antibody (Ki-67 Cell marque 275 R-1, dilution 1∶100 or DSG1/2 ProGen DG 3.10, dilution 1∶50) overnight at 4°C. The appropriate secondary antibody was applied for 120 min at room temperature. Nuclei were counter-stained with Hoechst 33258 and slides were mounted with fluorescence mounting medium (DAKO) and stored protected from light at 4°C until further analysis. Each IF staining was performed twice with the full set of slides (*n* = 12) on different days to ensure reproducibility. Unspecific IgG instead of primary antibody was used as negative control.

### Microscopic evaluation

Histological features in affected and control nasal biopsies were evaluated microscopically by one of the authors. For analysis and comparison of Ki67 between the two groups (affected versus non-affected dogs), the Ki67 positive cells in the basal layer and in total as well as the Hoechst 33258-positive cells in the basal layer were counted in five microscopic fields of each dog. Hyperproliferation (Ki67 basal : Hoechst 33258-positive cells basal), hyperplasia (Ki67 total : Hoechst 33258-positive cells basal), and the Ki67 basal : Ki67 total ratio were calculated.

### Statistical analysis

The data set of the IF staining results was analyzed using NCSS software (www.ncss.com) and applying multivariate analysis of variance (MANOVA). A *p*-value<0.05 was considered statistically significant.

## Supporting Information

Figure S1 Additional details on the phenotype of HNPK. (A) Photo of the nose of an HNPK affected Labrador Retriever illustrating the clinical symptoms. Note the crusts on the dorsal aspects of the nasal planum. (B) Non-affected control dog. Note the smooth, moist, and shiny nasal planum. (C) HE staining of a biopsy from the nose of an HNPK affected Labrador Retriever at 400× magnification illustrating the pronounced hyperkeratotic parakeratosis. Note the large number of retained nuclei in the stratum corneum. (D) Non-affected control dog. Note the complete absence of nuclei in the stratum corneum.
(TIF)
