# Supplementary figures and images for: A Mutation in the SUV39H2 Gene in Labrador Retrievers with Hereditary Nasal Parakeratosis (HNPK) Provides Insights into the Epigenetics of Keratinocyte Differentiation
Source: PLoS Genet. 2013 Oct 3;9(10):e1003848. doi: 10.1371/journal.pgen.1003848 (PMC3789836; doi:10.1371/journal.pgen.1003848)

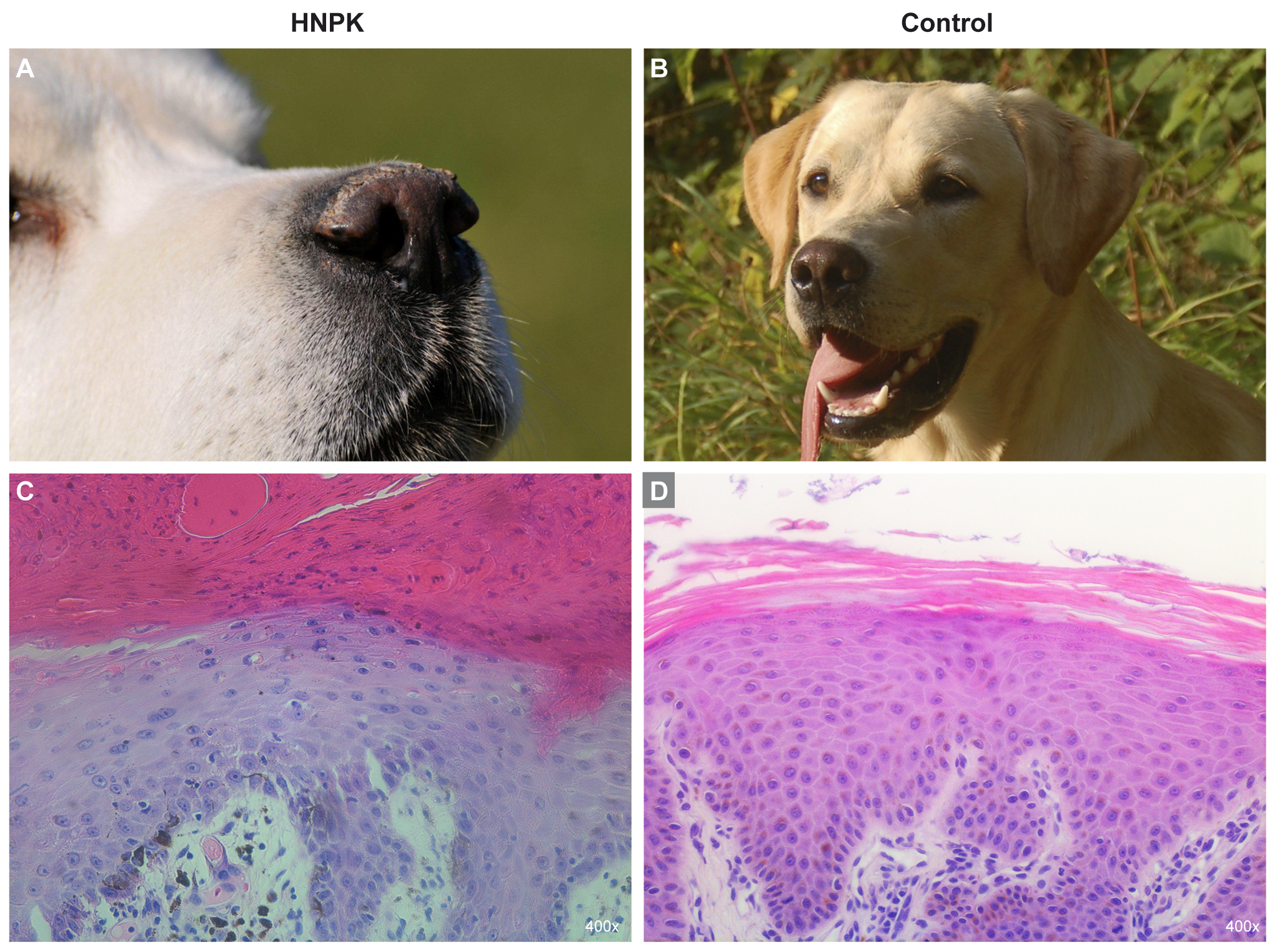

Supplement: Figure S1 — Additional details on the phenotype of HNPK. (A) Photo of the nose of an HNPK affected Labrador Retriever illustrating the clinical symptoms. Note the crusts on the dorsal aspects of the nasal planum. (B) Non-affected control dog. Note the smooth, moist, and shiny nasal planum. (C) HE staining of a biopsy from the nose of an HNPK affected Labrador Retriever at 400× magnification illustrating the pronounced hyperkeratotic parakeratosis. Note the large number of retained nuclei in the stratum corneum. (D) Non-affected control dog. Note the complete absence of nuclei in the stratum corneum. (TIF) [file pgen.1003848.s001.tif]
